# Outcomes of Electrosurgery Combined With 50% TCA Application in Patients With Rhinophyma

**DOI:** 10.1155/drp/4957519

**Published:** 2026-03-24

**Authors:** Gulsen Akoglu, Ahmet Tecik

**Affiliations:** ^1^ Department of Dermatovenereology, Gülhane Training and Research Hospital, University of Health Sciences, Ankara, Türkiye, akdeniz.edu.tr

**Keywords:** electrosurgery, rhinophyma, trichloroacetic acid

## Abstract

**Introduction:**

Rhinophyma causes significant cosmetic problems and social stigmatization. This study aimed to evaluate outcomes of electrosurgery combined with 50% trichloroacetic acid (TCA) treatment.

**Methods:**

The medical files and photographs of six patients who underwent rhinophyma electrosurgery combined with 50% TCA application were retrospectively analyzed. Demographic and clinical features of the patients, rhinophyma severity, posttreatment wound care, and treatment outcomes were reviewed. The cosmetic outcomes were evaluated with the physician’s cosmetic evaluation scale and the patient’s cosmetic satisfaction scale.

**Results:**

All six patients were male, with a mean age of 66.5 ± 3.5 years and a disease duration ranging from 3 to 21 years. The RHISI scores were 4 in two patients and 5 in four patients. All patients tolerated the whole process well. After electrosurgery, bismuth subgallate (BS) powder was poured for wound care. The BS coat remained until it dried and detached spontaneously from the skin after approximately 10 days. Subsequently, collagen gel was applied twice daily for 7–17 days. The mean time from the procedure day to the day of complete re‐epithelialization was 24.0 ± 4.7 days. Only one patient had marked scarring on alar regions, one had no complications, and the remaining four had only minimal, nondisturbing skin changes in limited areas. Patients’ cosmetic satisfaction scores ranged from 7 (satisfied) to 10 (extremely satisfied).

**Conclusion:**

Electrosurgery combined with 50% TCA application may provide a practical, well‐tolerated, and low‐cost treatment option in selected patients with rhinophyma. Postoperative wound care with topical BS powder and collagen gel serves a postoperative healing period of approximately three to 4 weeks.

## 1. Introduction

Rhinophyma is a subtype of rosacea characterized by nasal tissue deformity due to sebaceous gland hypertrophy, increased vascular structures, and connective tissue proliferation. Rhinophymatous changes usually occur in the 5th to 7th decades. The disorder is 5–30 times more common in men, probably related to the effect of androgens [1]. Patients usually seek treatment because of the social stigmatization caused by the significant cosmetic problems.

Medical treatments can only be partially effective in the early stages. Although radiotherapy was used successfully in treating rhinophyma in the 1920s, this method was abandoned due to the risk of secondary malignancy. Today, rhinophyma treatment is based on destructive procedures aiming to reduce the overgrown tissue volume. Treatment options are cryosurgery, dermabrasion, scalpel excision, electrosurgery, electrocautery, radiofrequency, laser ablation, and chemical peels applied alone or in combination [2]. Combination therapies are frequently employed, especially in moderate to severe cases, to enhance treatment efficacy and cosmetic outcomes.

Electrosurgery serves reasonable bleeding control, short surgical time, and easy application by cutting tissue and coagulating functions using high‐frequency alternating electric current. On the contrary, adjusting the excision depth is challenging and thermal damage may result in cartilage necrosis and the risk of scar formation [2]. Trichloroacetic acid (TCA) with low and high concentrations have been solely applied or combined with dermabrasion and electrosurgery in the treatment of rhinophyma [3–5]. However, the results of electrosurgery combined with TCA and long‐term follow‐up data are scarce in the literature. In this report, we detailed the outcomes of electrosurgery combined with 50% TCA treatment performed in 6 patients with rhinophyma and reviewed the literature.

## 2. Materials and Methods

The medical files and photographs of 6 patients who underwent rhinophyma electrosurgery combined with 50% TCA application between 2023 and 2024 in our dermatology clinic were retrospectively analyzed.

Demographic and clinical features of the patients, rhinophyma severity, posttreatment wound care, and treatment outcomes were reviewed. Rhinophyma Severity Index (RHISI) [6] at baseline was noted (no lesion = 0, mild skin thickening = 1, moderate skin thickening = 2, strong skin thickening with small lobules = 3, lobules with fissures = 4, and giant rhinophyma = 6; adding maximum one extra point if strong asymmetry, multiple cysts, or strong vessels is present).

### 2.1. The Procedure

**FIGURE 1:**
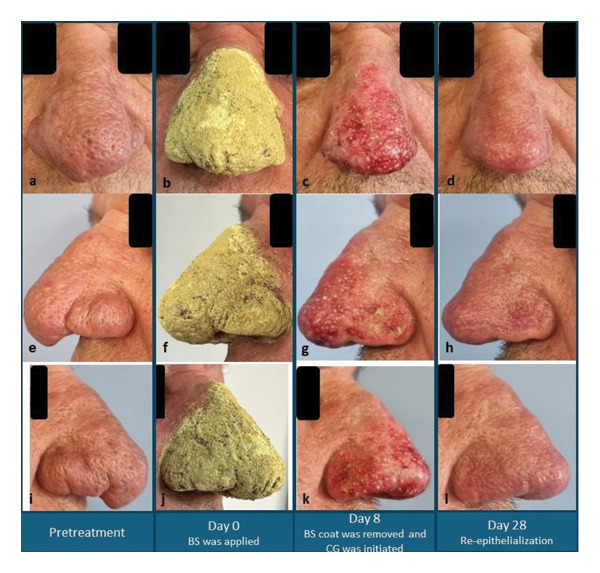
Patient 1; (a, e, i) pretreatment, (b, f, j) Day 0, when bismuth subgallate (BS) powder was applied; (c, g, k) Day 8, when BS was removed and collagen gel (CG) was initiated twice daily; (d, h, l) Day 28, complete re‐epithelialization was achieved.

All patients gave written informed consent before treatment and underwent only a single session. In all patients, a careful clinical and dermoscopic examination of the phymatous tissue was performed before treatment. No suspicious lesion suggesting malignancy was seen in any of the cases; therefore, biopsy was not considered necessary. Since all patients had a history of recurrent herpes labialis, herpes prophylaxis (valacyclovir 500 mg twice daily) was initiated 2 days before treatment and continued until complete re‐epithelialization to cover the period of greatest clinical susceptibility of the denuded facial wound surface. Before the intervention, petroleum jelly was used to protect the nasal mucosa and periocular area. Local anesthesia was performed by nerve blocks with lidocaine 2% with adrenaline, and infiltrative tumescent anesthesia was performed for soft tissue expansion to facilitate proper resection. The electrosurgery was performed in cutting–coagulation mode at 35 W by the same electrosurgery device using diathermy knife and loop electrodes for debulking the phymatous tissue for all patients. Care was taken to avoid excessive tissue resection, coagulation, and thermal damage, especially over the alar and tip cartilage. After achieving a proper resection depth (observing residual minimal yellowish sebaceous glands) and tissue hemostasis, 50% TCA was applied with cotton‐tipped applicators until uniform frosting appeared. Immediately after the procedure, we applied a thin layer of mupirocin ointment only once and poured the BS powder on the ointment as a thick layer and left in place. In the following days, the BS powder absorbed the exudate and covered the entire treated area as a drying dressing. The BS coat was removed when it remarkably dried and detached spontaneously. Afterward, the patients applied topical collagen gel (CG) containing 95% native collagen Type I and Type III extracted from bovine Achilles tendon (Regeneraty Collagen Gel, Medbiotec, Ankara, Türkiye) twice daily until re‐epithelialization was completed (Figure 1).

### 2.2. Evaluation of Cosmetic Outcomes

Secondary bacterial infections or herpes infection were not observed. The authors evaluated the cosmetic outcome of the procedure based on comparing the pretreatment baseline clinical photographs and posttreatment photos of complete re‐epithelialization. The physician’s cosmetic evaluation score was pointed on a scale of 0–10 and graded in four categories (*poor* = 0–3, *moderate* = 4‐6, *good* = 7–9, and *excellent* = 10). Patients’ scores of posttreatment cosmetic satisfaction on a scale of 0–10 were reviewed from their medical records, and their satisfaction was graded in four grades: disappointed, unsure, satisfied, and extremely satisfied. Adverse effects such as hypopigmentation, erythema, scarring, or atrophy were also noted.

## 3. Results

All patients were male with a mean age of 66.5 ± 3.5 years. Rhinophyma duration had a wide range, from 3 years to 21 years (mean ± SD = 13.3 ± 8.1 years) (Table 1). The RHISI scores were 4 in two patients and 5 in four (Table 2).

**TABLE 1:** Demographic characteristics of patients with rhinophyma.

Patient no	Age	Gender	Smoking status	Alcohol consumption	Family history	Duration (years)	Onset age (years)	Previous treatments
1	61	M	Active smoker	+	—	11	50	Oral isotretinoin, doxycycline, dermabrasion + TCA peel
2	70	M	Ex‐smoker	—	+ (father)	21	50	—
3	70	M	Ex‐smoker	Former drinker	—	5	65	Laser ablation with Er‐YAG
4	65	M	Ex‐smoker	Former drinker	—	20	45	Oral isotretinoin
5	68	M	Active smoker	+	+ (cousin)	3	65	—
6	65	M	Active smoker	—	+ (uncle)	20	45	—

*Note:* TCA: trichloroacetic acid.

**TABLE 2:** Clinical follow‐up of patients.

Patient no	Pretreatment RHISI score	Posttreatment wound care	Days to complete re‐epithelialization	Adverse effects	Follow‐up period (months)
1	5	8 days BS, then 17 days CG	25	Minimal atrophy on alar regions	4
2	5	12 days BS, then 7 days CG	19	—	6.5
3	5	13 days BS, 17 days CG	30	Minimal linear scarring on dorsum	5.5
4	4	18 days BS, then 11 days CG	29	Scarring on alar regions	6.5
5	5	5 days BS, then 15 days CG	20	Minimal linear scarring on right alar region	3
6	4	9 days BS, then 12 days CG	21	Minimal small hypopigmentation over dorsum	3

*Note:* BS: bismuth subgallate powder; RHISI: Rhinophyma Severity Index.

Abbreviation: CG: collagen gel.

**FIGURE 2:**
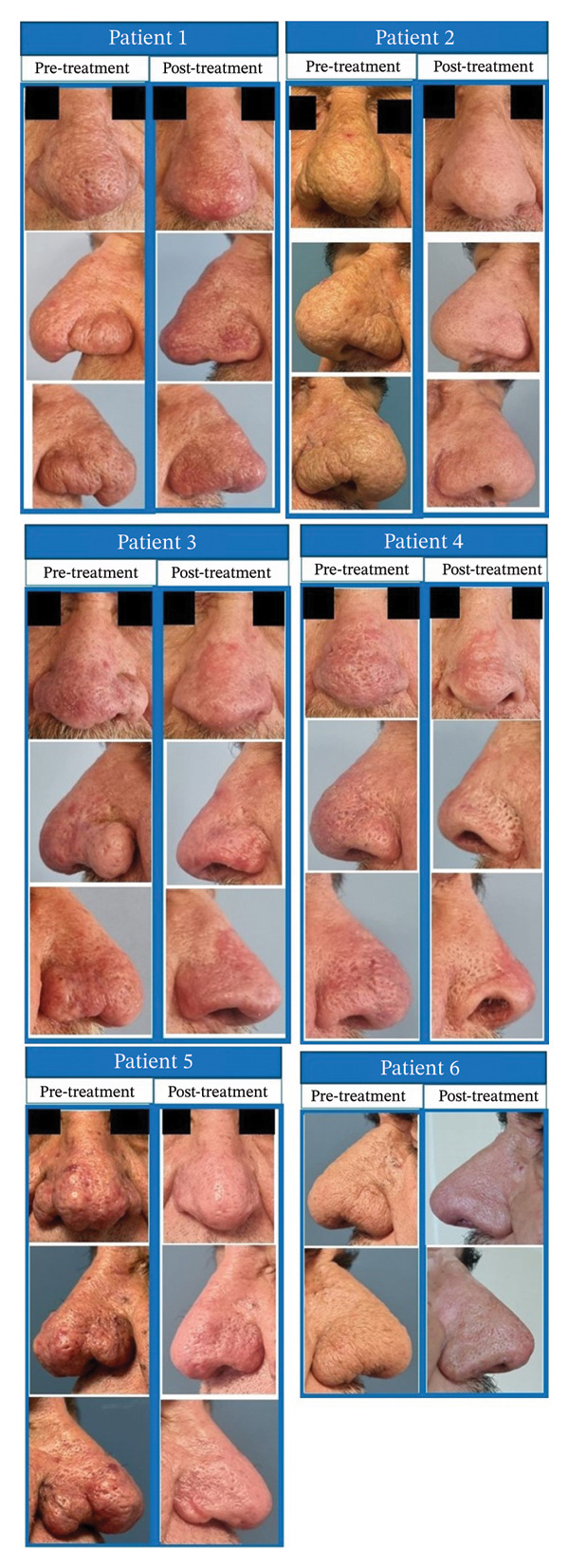
Clinical photographs of all patients showing pretreatment and posttreatment views.

All patients tolerated the whole process well. BS powder was poured as a coat on all patients when the procedure was completed. The BS coat remained until it dried and detached from the skin in 5–18 days (mean ± SD = 10.8 ± 4.5 days). Afterward, CG was applied to the noses of the patients twice daily. The mean needed duration of CG application was 13.2 ± 3.9 days (range = 7–17 days). The total duration from the procedure day (Day 0) to the day of complete re‐epithelialization took 19–30 days (mean ± SD = 24.0 ± 4.7 days). Only one patient had remarkable scarring (Patient 4) on alar regions; one had no complications, and the remaining four had minimal, nondisturbing skin changes (Figure 2). The cosmetic satisfaction scores of patients ranged between 7 (*satisfied*) and 10 (*extremely satisfied*), and almost all stated that they recommended the procedure to other patients complaining of rhinophyma. The cosmetic evaluation score of the physician ranged from 6 (*moderate*) to 9 (*good*) (Table 3). Patients were followed up for 3–6.5 months (mean ± SD = 4.8 ± 1.6 months). No additional skin changes or recurrences were observed.

**TABLE 3:** Patient satisfaction and physician’s cosmetic evaluation after treatment.

Patient no	Patient’s satisfaction score^∗^	Patient’s satisfaction grade^∗∗^	Does the patient recommend the procedure to other patients?	Physician’s cosmetic evaluation score^∗^	Physician’s cosmetic evaluation score^∗∗∗^
1	10	Extremely satisfied	Extremely recommends	8	Good
2	10	Extremely satisfied	Extremely recommends	9	Good
3	9	Extremely satisfied	Extremely recommends	9	Good
4	10	Extremely satisfied	Extremely recommends	6	Moderate
5	10	Extremely satisfied	Extremely recommends	8	Good
6	7	Satisfied	Recommends	9	Good

^∗^On a scale of 0–10.

^∗∗^Grade in 4 categories: disappointed, unsure, satisfied, and extremely satisfied.

^∗∗∗^Grade in 4 categories: poor (scores of 0–3), moderate (scores of 4–6), good (scores of 7–9), and excellent (score: 10).

## 4. Discussion

In the present study, we observed clinically and cosmetically satisfactory results with electrosurgery and 50% TCA treatment. In our cases, TCA application provided hemostasis by coagulation, as reported in the literature [3, 5]. Its positive effect was the destruction of phymatous tissue over challenging areas that are difficult to remove with electrosurgery. The application of TCA denatured the residual tissue, where there is concern about potentially excessive thermal damage by electrosurgery. Therefore, 50% TCA after electrosurgery provides hemostasis by its coagulation effect and helps remove residual phymatous tissue over areas risky for thermal damage and scarring. Indeed, caution is needed when applying TCA on extensively excised areas since TCA itself can cause remarkable or minimal scarring, as we have experienced in some of our patients. In only one patient, the developing hypertrophic scar on the alar region pulled the nostril toward itself, causing it to appear relatively wider. Thermal damage may have caused scarring, but applying TCA on top of it may have resulted in more intense scarring. Therefore, TCA application should be used on limited areas demonstrating more obvious residual tissue to be removed and not applied over deeply excised areas, especially when over cartilage. In addition, the uniform, dense frosting should be the limit of security; “camel color” points out that scarring will develop in that area.

Applying 35% TCA for mild rhinophyma and higher concentrations up to 70%–90% TCA for severe cases have been reported to achieve remarkable outcomes even 7–10 days of healing after a single session [4]. When compared, our cases also had severe rhinophyma; we preferred electrosurgery and then TCA application to remove the lobulated firm tissues, which did not seem to be able to be removed by solely a single application of high TCA concentration. Indeed, to make an objective evaluation, studies comparing both methods are needed. By any method other than electrosurgery, Fırat et al. applied 45% TCA after deep dermabrasion and observed minimal bleeding, controlled tissue excision, and minimal to moderate exfoliation. The coagulative effect of the TCA application facilitated the dermabrasion process [5].

Treatment modalities used for rhinophyma have various advantages and disadvantages. A good hemostasis and controlled depth of tissue removal are the main targeted endpoints that avoid adverse effects. Both electrosurgery/electrocautery and CO_2_ laser ablation serve good hemostasis, but cost‐effectiveness may be a major issue for laser [2]. Reviewing experiences on surgical treatment of rhinophyma, Dogourd et al. recommended cold blade excision and application of 30% TCA to promote coagulation for mild cases and monopolar diathermy knife for severe ones [3]. A clearer view of the excision area is the main advantage of the CO_2_ laser. Although the thermal damage is lesser than the electrosurgical approach, the risk of scarring and pigmentation changes can occur, especially on alar regions or the tip of the nose [2, 3].

Wound care after rhinophyma treatment is critical, especially for older patients and ones with comorbidities. A rapid re‐epithelialization period without any infection is desired. Bismuth subgallate is a yellow powder with hemostatic and astringent properties and serves a broad‐spectrum antibiotic activity against Gram‐negative and positive bacteria [7, 8]. The reports about using BS in wound healing have conflicting results, and a rat study demonstrated that BS does not affect inflammatory response, collagen production, and angiogenesis [7]. We used BS powder as a low‐cost, easily applicable astringent hemostatic and protective coat in the early postoperative period. Besides, the patients did not touch the area for applying any topical agent, avoiding secondary bacterial infections. Within a week, the powder completed its action and dried, and then, BS coat was removed, and the residual wound erosions were covered with CG to promote wound healing. The CG provided complete re‐epithelialization healing in one to 2 weeks. We suggest that the consecutive application of BS powder and CG facilitates hemostasis and wound healing within a period that patients can easily tolerate. Furthermore, this kind of wound care costs less when compared with expensive wound dressings and topical wound care products, which are usually applied more frequently.

## 5. Limitations

Although our study demonstrated favorable outcomes in both patients’ and physicians’ cosmetic evaluations, it involved a very limited number of patients. Cosmetic outcomes were assessed using nonvalidated and subjective scales; in addition, physician ratings were performed by the authors and were not blinded, which may have introduced observer bias. To confirm our findings and draw more robust conclusions, further studies on larger patient groups that compare our procedure with other techniques through objective cosmetic evaluations and assess the long‐term stability of the results are necessary.

## 6. Conclusions

Electrosurgery combined with 50% TCA application followed by a healing period of three to 4 weeks by simple wound care with topical BS powder and CG, may be a practical, tolerable, and low‐cost treatment option in selected patients with rhinophyma. Care should be taken to avoid complications when removing the tissues over cartilage and applying TCA over thinned areas.

## Author Contributions

Gulsen Akoglu and Ahmet Tecik designed the research study. Gulsen Akoglu performed the procedures, and Ahmet Tecik assisted during the procedure and follow‐ups of the patients. Ahmet Tecik contributed essential reagents or tools. Gulsen Akoglu wrote the manuscript.

## Funding

Medbiotec (Ankara, Türkiye) provided Regeneraty Collagen Gel free of charge for patient care.

## Disclosure

The company had no role in study design, data collection, analysis, manuscript preparation, or decision to submit. All authors have read and approved the final manuscript.

## Ethics Statement

The study was conducted according to the principles of the Helsinki Declaration and was approved by Gülhane Training and Research Hospital Ethics Committee (Approval Date and Number: 10/04/2025, #2025/47‐rev.) for a retrospective review of medical records and clinical photographs. All patients gave written informed consent for the procedure and publication of any potentially identifiable images.

## Conflicts of Interest

The authors declare no conflicts of interest.

## Data Availability

The data that support the findings of this study are available from the corresponding author upon reasonable request.
